# Protocol for comparing gene-level selection on coding mutations between two groups of samples with *Coselens*

**DOI:** 10.1016/j.xpro.2023.102117

**Published:** 2023-02-14

**Authors:** Jaime Iranzo, George Gruenhagen, Jorge Calle-Espinosa, Eugene V. Koonin

**Affiliations:** 1 Centro de Biotecnología y Genómica de Plantas, Universidad Politécnica de Madrid (UPM) - Instituto Nacional de Investigación y Tecnología Agraria y Alimentaria (INIA), Madrid, Spain; 2 Institute for Biocomputation and Physics of Complex Systems (BIFI), University of Zaragoza, Zaragoza, Spain; 3 Institute of Bioengineering and Biosciences, School of Biological Sciences, Georgia Institute of Technology, Atlanta, GA, USA; 4 National Center for Biotechnology Information, National Library of Medicine, National Institutes of Health, Bethesda, MD, USA

**Keywords:** Bioinformatics, Cancer, Evolutionary biology, Genomics

## Abstract

The study of genes that evolve under conditional selection can shed light on the genomic underpinnings of adaptation, revealing epistasis and phenotypic plasticity. This protocol describes how to use the *Coselens* package to compare gene-level selection between two groups of samples. After installing *Coselens* and preparing the datasets, a typical run on a laptop takes less than 10 min. *Coselens* is best suited to analyze somatic mutations and data from experimental evolution, for which independently evolved samples are available.

For complete details on the use and execution of this protocol, please refer to Iranzo et al. (2022).^1^

## Highlights

• *Coselens* quantifies gene-level conditional selection based on coding mutations
• Analyze medium-size datasets on a laptop in less than 10 min
• Input mutation tables from whole-genome or targeted sequencing
• Output genes subject to conditional selection, effect sizes, and p values

## Abstract (editor highlights)

Publisher’s note: Undertaking any experimental protocol requires adherence to local institutional guidelines for laboratory safety and ethics.

## Graphical abstract

**Figure:**
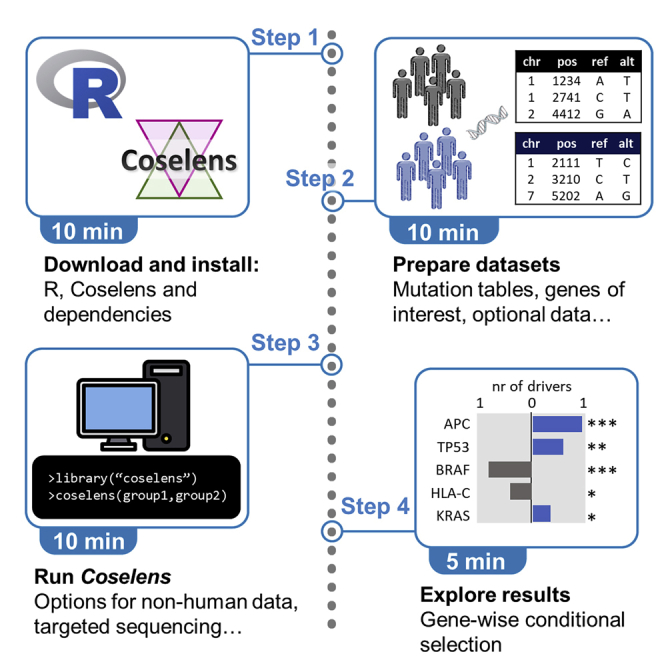

## Before you begin

COnditional SELection on the Excess of NonSynonymous Substitutions (*Coselens*) is an R package to detect gene-level differential selection between two groups of samples. If the samples are grouped based on the value of a binary variable (e.g., the presence or absence of some environmental stress or phenotypic trait), *Coselens* identifies genes that harbor conditional driver mutations (that is, mutations whose adaptive value depends on the grouping variable). *Coselens* provides maximum likelihood estimates of the average number of driver mutations per gene per sample by comparing the observed number of coding (nonsynonymous and truncating) substitutions and indels with the number of mutations that would be expected in the absence of selection. *Coselens* also classifies instances of conditional selection based on their sign and the magnitude of their association with the independent variable. A major application of *Coselens* is to infer epistasis networks that can be later analyzed with appropriate tools to extract relevant biological information. The workflow to generate epistasis networks with *Coselens* is explained in the section quantification and statistical analysis.

*Coselens* uses the *dndscv* R package^2^ to estimate a neutral mutation model that takes into account trinucleotide contexts and genomic covariates. As a result, *Coselens* is subject to the same assumptions as *dndscv*. Although explaining the use of *dndscv* is out of the scope of this protocol, we describe its critical assumptions in the data preparation and troubleshooting sections. *Coselens* also admits most of the optional parameters of *dndscv*. The most relevant of these parameters are described along the protocol.

This section specifies the system requirements, installation instructions, and file formats required by *Coselens*. All the commands included in this protocol are intended to be run inside an interactive R session. Proficiency in R is not required to conduct simple analyses with *Coselens*. However, some degree of familiarity with R scripting may be useful to perform high-throughput analyses and to integrate *Coselens* into more complex workflows.

### System requirements

Hardware: small and medium-size datasets (up to around 1M mutations per group) can be processed by a laptop with 8GB RAM. More memory may be needed to run *Coselens* in larger datasets.

Software: The R software environment for statistical computing must be installed in the system. *Coselens* has been tested with R version 4.0.0 and newer. The exact instructions to obtain and install R depend on the operative system. For more details, we refer the reader to the R Project site (https://www.r-project.org/).

### Installation of *Coselens* and its dependencies

**Timing: 10 min**

1. *Coselens* depends on the following R packages: devtools, geometry, seqinr, GenomicRanges, Biostrings, IRanges, and MASS. Except for devtools, which should be manually installed before *Coselens*, all the required dependencies are automatically installed when the user installs *Coselens.* To check if devtools is already installed and install it otherwise, run the following command:

>if (!require("devtools")) install.packages("devtools")

2. Use the following command to install the latest version of *Coselens* from the GitHub repository:

>library("devtools")

>devtools::install_github("ggruenhagen3/coselens")

A successful installation will return no errors and the last printed line will say “Done”. If automatic installation of dependencies fails, see troubleshooting 1.

***Optional:****Coselens* makes extensive use of the *dndscv* R package.^2^ A slightly customized version of this package is included with Coselens and runs under-the-hood. However, for some particular applications (e.g., to analyze non-human data), it may be helpful to install the original version of *dndscv* by running the following command:

>devtools::install_github("im3sanger/dndscv")

### Data preparation

**Timing: 10 min**

*Coselens* infers conditional selection at the gene level by comparing mutational profiles between two datasets. One dataset is built from samples that are subject to the condition of interest; the other, treated as control, includes samples that do not meet that condition.

The only mandatory input for *Coselens* consists of two data frames with the mutations observed in the condition and the control groups. The easiest way to provide such information is by compiling two separate mutation tables as described below. Additional input files allow filtering the results according to a list of genes of interest or extending the basic functionality of *Coselens* beyond the most usual applications.

**Figure 1:**
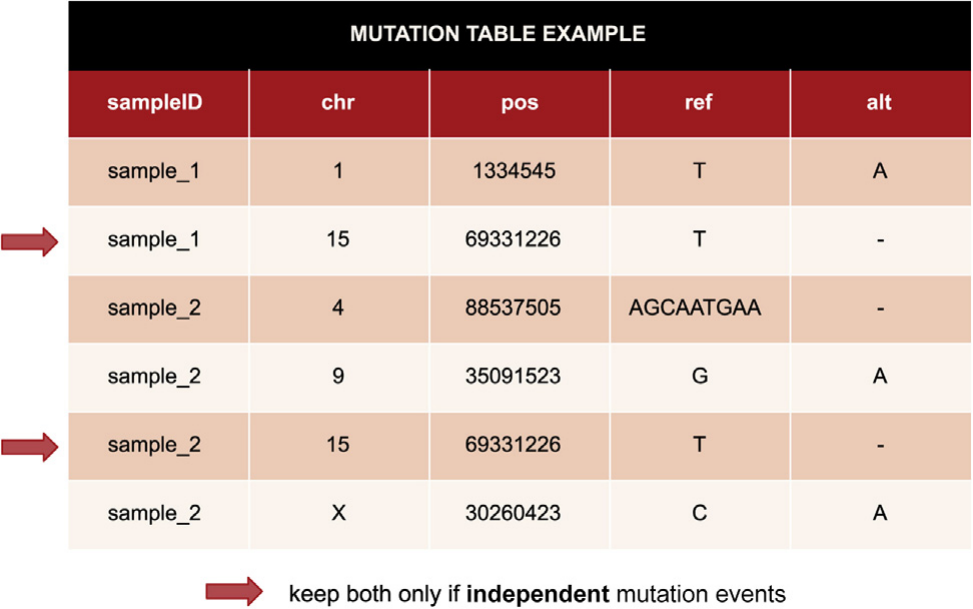
Example of a mutation table that could be an input for *Coselens* (only 6 rows are shown) The arrows indicate two mutations in the same position but in different samples. Redundant mutations like those should only be kept if they represent independent mutation events.

3. Prepare two mutation tables, one for each group of samples. Each table should have 5 columns and as many rows as mutations (Figure 1). For each mutation, the columns indicate the sample in which it was found (sampleID), the chromosome (chr) and position (pos) of the mutation within the chromosome, the reference base (ref), and the mutated base (alt). The columns should follow that exact order. The mutation tables can be stored as R data frames or text files with columns delimited by commas or tabs.

**CRITICAL:** It is important that each mutation listed in the table represents an independent mutation event. If multiple instances of the same mutation derive from a single mutation event (for example, when sequencing related samples from the same individual), only one record should be kept in the table. In that case, the mutation can be arbitrarily assigned to any of the samples that harbor it (see troubleshooting 2 for more details). However, if a recurrent mutation is present in different samples as a result of independent mutation events (for example, somatic mutations affecting a known mutational hotspot in different individuals), all instances should be included.

***Note:*** The mutational model estimated by *Coselens* does not account for double mutations or mutational signatures spanning more than 3 nucleotides. Although generally rare, those can be relevant, for example, in ultrahypermutator tumors. Therefore, to avoid inaccurate estimates, it is generally safer to remove heavily mutated samples from the dataset. Two broadly used criteria to identify ultrahypermutators are the following: (a) >3,000 coding mutations per exome; (b) >3 standard deviations in the total number of mutations with respect to all samples.

***Note:*** To maximize the accuracy and sensitivity of *Coselens*, we strongly recommend that all sequenced genes are considered when preparing the mutation tables. A list of genes of interest can be separately provided to *Coselens* (see below) to filter the results.

**CRITICAL:** By default, *Coselens* assumes that the mutation data is mapped to the GRCh37/hg19 assembly of the human reference genome. To use *Coselens* with different species or assemblies, or to work at genomic resolutions different than the gene level, an alternative reference database (RefCDS object) must be provided with the option refdb (see running Coselens – step 4). The generation of alternative reference databases can be done using the buildref function in the *dndscv* package (see troubleshooting 3 for more information). Alternatively, CrossMap^3^ can be used to convert mutation coordinates across assemblies of the human genome.

4. Prepare additional input files.
  a. List of genes of interest (optional). If provided, the results and Benjamini-Hochberg corrections will be restricted to those genes. The file can be formatted as a 1-column or 1-row (comma-delimited) list, with no headers.
  b. List of sequenced genes (not required for whole-exome or whole-genome sequencing data). This is mandatory if working with targeted sequencing data. The file can be formatted as a 1-column or 1-row (comma-delimited) list, with no headers.
  c. Alternative reference database (RefCDS object) and covariate table. Only needed if working with non-human data or assemblies other than the GRCh37/hg19. Covariates can also be used to account for cross-gene differences in sequencing coverage and other potential confounding factors, such as structural variants or copy-number variants. See troubleshooting 3 for more information on how to generate custom reference databases and covariate tables.

## Key resources table

**Table:** 

REAGENT or RESOURCE	SOURCE	IDENTIFIER
**Deposited data**		

Example dataset - TCGA somatic mutation calls for colorectal cancer	Ellrott et al.^4^; Multi-Center Mutation Calling in Multiple Cancers, NIH Genomic Data Commons.	https://gdc.cancer.gov/about-data/publications/mc3-2017; file mc3.v0.2.8.PUBLIC.maf.gz
Example dataset - List of 441 previously reported cancer genes	Iranzo et al.^1^	https://www.cell.com/cms/10.1016/j.celrep.2022.111272/attachment/844802ad-e5bc-484a-b801-5906d231c25a/mmc2.xlsx

**Software and algorithms**		

Coselens R package	Iranzo et al.^1^	https://github.com/ggruenhagen3/coselens; RRID: SCR_022578; https://doi.org/10.5281/zenodo.7343763
dndscv R package	Martincorena et al.^2^	https://www.sanger.ac.uk/tool/dndscv/; RRID: SCR_017093
CrossMap	Zhao et al.^3^	https://crossmap.sourceforge.net/; RRID: SCR_001173
Cytoscape	Shannon et al.^5^	https://cytoscape.org; RRID: SCR_003032
NetworkX	NetworkX Developers	https://networkx.github.io; RRID: SCR_016864
igraph	CRAN	https://igraph.org/; RRID: SCR_019225
SiMap	Esmailian and Jalili^6^	https://github.com/pouyaesm/signed-community-detection

## Step-by-step method details

Here we describe how to conduct an analysis of conditional selection with *Coselens*. To illustrate how this works in practice, we use data from a recent study that investigated epistasis between driver mutations in cancer.^1^ The key idea is that epistatic interactions can be detected by looking for conditional selection on driver mutations. More precisely, epistasis implies that the effect of driver mutations in one gene depends on the presence or absence of driver mutations in another gene. Because the intensity of selection on a mutation is proportional to its fitness effect, epistasis makes selection for driver mutations in one gene conditional on the presence (or absence) of driver mutations in the other gene. Following this rationale, to identify genes that participate in epistatic interactions with the tumor suppressor gene *APC*, the authors of the study collected somatic mutation data from a large number of colorectal tumors and classified the tumors into two groups, depending on whether they harbored potential driver mutations in *APC* or not. Then, epistatic partners were identified with *Coselens* as genes showing evidence of differential selection between the two groups of tumors.

### Running Coselens

**Timing: 5–10 min (more for datasets with >100K mutations)**

To run *Coselens*, all the relevant data must be first loaded in R. Then, *Coselens* is executed with options set according to the origin of the data and the purpose of the analysis. We describe the most relevant options and their usage below.

1. Load the *Coselens* package (troubleshooting 1):

>library("coselens")

2. Load mutation tables as data frames.
  a. Identify the paths to each mutation table:
    >group1_file = "/path/to/table_file_1.csv"
    >group2_file = "/path/to/table_file_2.csv"
    ***Note:*** Replace /path/to/table_file_1.csv and /path/to/table_file_2.csv by the actual location of the files, keeping the double quotes.
  b. Load mutation tables. Comma-delimited files can be loaded with the following commands:
    >group1 = read.csv(group1_file, stringsAsFactors = FALSE)
    >group2 = read.csv(group2_file, stringsAsFactors = FALSE)
    ***Note:*** To load tab-delimited files, add the argument sep = "\t" when calling the function read.csv().
    ***Note:****Coselens* includes some example datasets that can be directly loaded and used for testing. To load the example data, run the following lines of code and skip step 3:
    >data("group1", package = "coselens") # mutations from colorectal tumors with drivers in APC
    >data("group2", package = "coselens") # mutations from colorectal tumors without drivers in APC
    >data("cancer_genes", package = "coselens") # list of cancer genes
    >sequenced_genes = cancer_genes # list of sequenced genes (same as list of cancer genes)
  c. After running step 2, there will be two data frames in the R workspace, named group1 and group2, that contain the mutations found in each group of samples. You can inspect the content of these data frames by running the R command head(). For example, to show the first lines of group1, run
    >head(group1)

***Note:*** If the file was correctly loaded, the output should coincide with the first lines of the original file.

3. Load the list of sequenced genes (for targeted sequencing data) and genes to include in the results report (optional).

>sequenced_genes_file = "/path/to/sequenced_genes_file.csv"

>sequenced_genes = read.csv(sequenced_genes_file, stringsAsFactors = FALSE, header=FALSE)

>unlist(as.vector(sequenced_genes))

>cancer_genes_file = "/path/to/cancer_genes_file.csv"

>cancer_genes = read.csv(cancer_genes_file, stringsAsFactors = FALSE, header=FALSE)

>unlist(as.vector(cancer_genes))

***Note:*** Replace /path/to/sequenced_genes_file.csv and /path/to/cancer_genes_file.csv by the actual locations of the files with the list of sequenced genes and the genes of interest, respectively, keeping the double quotes. Use header=TRUE if the file has headers.

***Note:*** In most cases involving targeted sequencing data, it makes sense to use the same list to indicate which genes were sequenced and which should be included in the results report. However, there are some scenarios in which using different lists could be desirable (for example, if reusing targeted sequencing data that covered genes that are not relevant to the current study).

***Note:*** After running step 3, the data frame cancer_genes will contain the list of genes of interest (that is, those that should be included in the results report), and sequenced_genes will contain the list of genes covered by targeted sequencing.

4. Run *Coselens* and filter the results to only include a subset of genes of interest (for example, genes involved in cancer initiation and progression).
  a. The following command would apply to whole-exome sequencing data mapped to the GRCh37/hg19 assembly of the human reference genome.
    >coselens_out = coselens(group1, group2, subset.genes.by = cancer_genes)
    ***Note:*** When using the subset.genes.by option, the Benjamini-Hochberg correction for false discovery rate is only applied to the filtered gene set. To show the results for all genes in the reference database (around 20k for human data) and apply the false discovery rate correction to the whole gene set, omit the option subset.genes.by = cancer_genes.
  b. To analyze data from targeted sequencing, use the optional parameter sequenced.genes to indicate the list of sequenced genes:
    >coselens_out = coselens(group1, group2, sequenced.genes = sequenced_genes, subset.genes.by = cancer_genes)
  c. To work with mutation data from other species or mapped to other assemblies of the human genome, use the optional parameter refdb to indicate the file that contains the alternative reference database.
    >coselens_out = coselens(group1, group2, refdb = "/path/to/ref_db_file.rda", cv = NULL)

***Note:*** By default, the covariates used by *Coselens* (more precisely, the *dndscv* function underneath *Coselens*) for statistical inference are specific to the GRCh37/hg19 assembly of the human genome. If working with other species or assemblies, the option cv = NULL should be included to instruct *Coselens* to not use covariates. Not using covariates can reduce the performance of the method, especially in datasets with large unexplained variation in the mutation rates across genes. Alternatively, if covariates are available for the species of interest, they can be used by setting cv = covariates, where covariates is a data frame with gene names as rows and one column per covariate. See troubleshooting 3 for further information on how to generate alternative covariate files.

***Note:*** During execution, the program will display several messages indicating its progress. Pay special attention to the values of θ (theta) in the regression models for substitutions and indels. Low values of θ, particularly θ < 1, indicate that there is large unexplained variation in the mutation density across genes so that the model used by *Coselens* might not be suitable for this dataset. For more information about possible warnings and errors produced in this step, see troubleshooting 2–8.

### Visualization and interpretation of the results

**Timing: 5 min**

*Coselens* returns several tables with effect sizes and p-values for differential selection in each gene of the reference genome. If a list of genes is provided through the subset.genes.by option, the results and Benjamini-Hochberg corrections are restricted to those genes. This section explains how to visualize, save, and filter the results based on statistical significance. The columns of the results table are described in the Expected Outcomes section.

**Figure 2:**
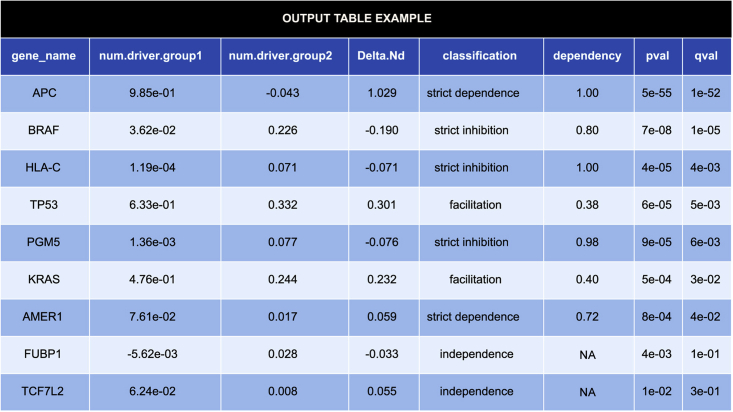
Example of an output table generated by *Coselens.* The contents of the table are described in detail in the Expected Outcomes section.

5. Pre-view and save the results of the analysis (gene-wise conditional selection based on coding substitutions).
  a. Pre-view the first rows of the results table, sorted by false discovery rate:
    >head(coselens_out$substitutions[order(coselens_out$substitutions$qval),])
    ***Note:***Figure 2 shows, as an example, a fragment of the output table generated by *Coselens*. The contents of the table are described in detail in the Expected Outcomes section.
    ***Note:*** Replace coselens_out$substituions by coselens_out$indels to explore differential selection on indels (see also the notes below step 6).
  b. Save the results table as a comma-delimited file:
    >write.table(coselens_out$substitutions, "/path/to/output_file.csv", sep = ",", quote = FALSE, row.names = FALSE)
    ***Note:*** Replace /path/to/output_file.csv by the location where the file should be saved, keeping the double quotes. To save as a tab-delimited file, replace sep="," with sep="\t".

6. Filter genes subject to statistically significant conditional selection on driver substitutions at a 5% false discovery rate:

>coselens_out$substitutions[which(coselens_out$substitutions$qval < 0.05),]

***Note:*** To filter results based on other criteria, replace qval by the name of the desired column (for example, pval for p-values) and 0.05 by the significance level.

***Optional:****Coselens* also looks for differential selection on indels and registers the separate contributions of missense and truncating (nonsense and essential splice site) substitutions to the total number of driver substitutions. Result tables for indels, missense substitutions, and truncating substitutions can be found in coselens_out$indels, coselens_out$missense_sub, and coselens_out$truncating_sub, respectively. *Coselens* also returns the results of a joint analysis of single-nucleotide substitutions and indels in the table coselens_out$overall_mut. In the latter case, p-values are obtained using Fisher’s formula for aggregated probabilities (troubleshooting 9). To pre-view, save, and filter these tables, repeat steps 5 and 6 and replace coselens_out$substitutios by the name of the table of interest.

***Note:****Coselens* uses different statistical frameworks to infer the number of driver substitutions and indels. The null model for substitutions is obtained by fitting the number of observed synonymous substitutions to a 192-rate model with gamma-distributed across-gene variability and genomic covariates. The null model for indels is based on the number of indels found in coding regions that do not belong to the set of genes of interest, accounting for gene length and genomic covariates. Therefore, any indel in a gene that does not belong to the set of genes of interest is considered neutral. In practice, these different approaches imply that the sensitivity of driver inference is often much higher for substitutions than for indels. As a result, caution must be taken when comparing results obtained for substitutions and indels.

***Note:*** Aggregated p-values calculated using Fisher’s formula, such as those provided for the combination of substitutions and indels in the table coselens_out$overall_mut, can be overly conservative if the tests that are being combined suffer from low sensitivity. In our experience with cancer somatic mutation data, the indel test (coselens_out$indels) only attains good sensitivity if the sample size is large (>100 samples) and indels are frequent. Otherwise, the low sensitivity of the indel test can result in non-significant p-values when jointly analyzing substitutions and indels (coselens_out$overall_mut), despite the presence of substantial differential selection on substitutions (troubleshooting 9). Thus, we recommend using the table coselens_out$substitutions to assess the significance of differential selection on substitutions, while restricting coselens_out$indels and coselens_out$overall_mut to datasets with large sample sizes and genes in which indels are the main subject of selection.

***Optional:****Coselens* returns coselens_out$dndscv, a list of objects with the complete output of (non-conditional) selection analyses separately run on each group of samples, as provided by the *dndscv* package. A possible application of these objects is to compare mutational signatures between groups, which can be done by using the maximum likelihood estimates of the mutation models found in coselens_out$dndscv$dndscv_group1$mle_submodel and coselens_out$dndscv$dndscv_group2$mle_submodel. For more detail on the structure and information contained in these objects, we refer the reader to the documentation of the *dndscv* package (https://github.com/im3sanger/dndscv).

## Expected outcomes

By completing this protocol, five output tables (coselens_out$substitutions, coselens_out$indels, coselens_out$missense_sub, coselens_out$truncating_sub, and coselens_out$overall_mut) and two *dndscv* objects (coselens_out$dndscv$dndscv_group1 and coselens_out$dndscv$dndscv_group2) will be generated.

The first table (coselens_out$substitutions, Figure 2) contains the average number of driver substitutions per gene for a list of genes of interest, separately estimated for each group of samples, a qualitative classification of the dependency between selection for drivers and the grouping variable, and two measures of effect size for differential selection (see details below). The table also reports the statistical significance of maximum likelihood tests assessing differences in the number of driver substitutions between groups. If the grouping variable refers to the presence or absence of a given experimental, environmental, or genomic condition, significant differences in the number of drivers reveal the existence of conditional selection. If groups are made based on the presence or absence of coding mutations in a gene of interest, searching for differential selection between groups identifies genes that are involved in epistasis with that gene. This table should be sufficient for most users. Regarding the interpretation of the results, conditionality can be caused by changes in the phenotypic effect of mutations or in the selective pressures, whenever such changes correlate with the grouping variable. However, because *Coselens* corrects for differences in the mutational background, changes in mutation rates and mutational mechanisms can be ruled out as causes for the inferred instances of conditional selection.

The first column of the results table (gene_name) corresponds to the name of the gene. The values in columns 2 and 3 (num.driver.group1 and num.driver.group2) are the estimates of the excess of non-synonymous substitutions with respect to the neutral expectation in groups 1 and 2, respectively. Non-synonymous substitutions include missense, nonsense, and essential splice site mutations. In the absence of negative (purifying) selection, as is the case of most tumors,^2^^,^^7^ the excess of mutations in a gene coincides with the average number of drivers (that is, positively selected mutations) per sample in that gene.

**Figure 3:**
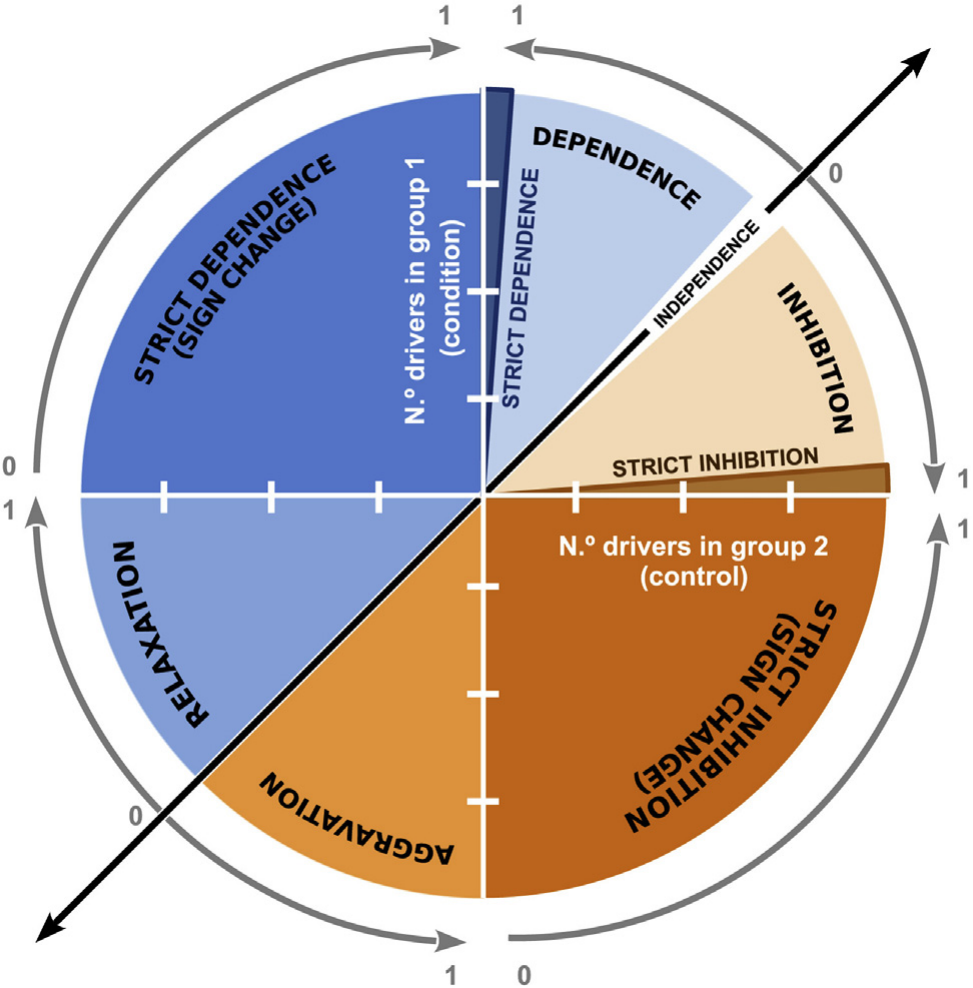
Dependency index and classes of conditional selection Instances of conditional selection are classified by comparing the number of drivers when the condition is met (group 1, y-axis) and when it is not met (group 2, x-axis). Genes unaffected by differential selection are located on the diagonal (independence). Genes that only harbor driver mutations when the condition is met (strict dependence) or when it is not met (strict inhibition) appear on or near the positive y- and x-axis, respectively. Gray arrows from 0 to 1 indicate the ranges of the dependency index for each class of conditional selection.

A major feature of *Coselens* is that it provides the user with biologically meaningful effect sizes for the magnitude of conditional selection. The most straightforward way to quantify effect sizes is by calculating the difference in the average number of driver mutations in the presence and absence of the condition of interest. We call that measure of effect size Δ*N*_*d*_ (column 4, Delta.Nd). The value of Δ*N*_*d*_ indicates, in absolute terms, how the grouping variable modifies the average number of driver mutations in a gene. Column 5 (classification) provides a qualitative classification of the association between the grouping variable (the condition of interest) and the magnitude and sign of selection (Figure 3). *Independence* implies that the grouping variable does not affect selection for drivers; *strict dependence* implies that drivers are only positively selected in the first group of samples, in which the condition of interest is met; *strict inhibition* implies that positive selection only acts in the second group, in which the condition is not met. Strict dependence and strict inhibition are the two extremes of conditionality, that is, cases of full conditionality. Instances of partial conditionality are labeled as *facilitation* and *inhibition*, respectively. Together with independence, these four classes cover the whole spectrum of dependencies for positively selected driver mutations. If negative (purifying) selection is dominant, other classes of dependency become possible: *strict dependence with sign change*, if the sign of selection changes from negative to positive when the condition is met; *strict inhibition with sign change*, if the sign of selection changes from positive to negative when the condition is met; *aggravation*, if purifying selection against mutations becomes stronger when the condition is met; and *relaxation*, if selection against mutations becomes weaker when the condition is met. A quantitative measure of these associations is given by the dependency index (column 6, dependency), which takes values between 0/NA (no conditionality) and 1 (full conditionality). Note that some instances of strict dependence and strict inhibition display dependency indices close (but not exactly equal) to 1. The reason is that strict dependence and strict inhibition are classified as such if the average number of drivers in one of the groups is not significantly greater than zero (but not necessarily exactly equal to zero).

The last two columns (pval and qval) report the statistical significance and false discovery rate of differential selection, obtained through a likelihood ratio test as described in Iranzo et al*.*^1^ The false discovery rate is obtained by applying the Benjamini-Hochberg correction for multiple testing to all the genes shown in the table.

The additional tables coselens_out$indels, coselens_out$missense_sub, coselens_out$truncating_sub, and coselens_out$overall_mut summarize the results of the differential selection analysis for indels, missense substitutions, truncating (nonsense and splice site) substitutions, and the combination of indels and single nucleotide substitutions. The columns of these tables are analogous to those in coselens_out$substitutions. Note that the analysis of indels uses a different null model that makes the test for differential selection notably less sensitive than in the case of substitutions. Such lower sensitivity also extends to the combined analysis of substitutions and indels (table coselens_out$overall_mut).

The objects coselens_out$dndscv$dndscv_group1 and coselens_out$dndscv$dndscv_group2 store the complete output of the selection analysis tool *dndscv* separately run on each group of samples. Advanced users can use these objects to study differences in mutational signatures and mutation rates, and to extract additional information not included in the main output tables.

The protocol allows for some degree of flexibility to accommodate the needs of the user. Although it is easiest to use with human data, it can be applied to other species by generating or downloading reference genome databases for the species of interest.

## Quantification and statistical analysis

In this section, we provide general guidelines on how to use *Coselens* to obtain epistasis networks and suggest external software for network analysis.

• The first step is to identify candidate genes that are potentially involved in epistasis. To that end, we recommend looking for genes subject to significant positive selection using the *dndscv* R package.
• The following steps are separately performed for each candidate gene and involve splitting the mutation dataset in two groups, one including all the samples that harbor coding mutations in the candidate gene, the other including the rest of the samples. More stringent criteria can be applied to ensure that the splitting corresponds to the presence or absence of potential driver mutations (instead of just coding mutations) in the candidate gene. For example, the user may want to look for samples harboring truncating mutations in known tumor suppressor genes or substitutions hitting mutational hotspots in oncogenes. Then, both groups of samples are compared with *Coselens.* To increase the statistical power, we recommend setting the option subset.genes.by to restrict the false discovery rate correction to a list of genes of interest (for example, known cancer genes or genes subject to significant positive selection). Finally, the results table (coselens_out$substitutions) is filtered to keep only the rows with significant q-values, a new column is added to the left with the name of the candidate gene, and columns num.driver.group1, num.driver.group2, and dependency are dropped. Advanced users may want to automatize and parallelize these steps by running one independent process for each candidate gene.
• In the final step, the tables obtained for each candidate gene are merged. Each row of the merged table corresponds to an edge or link of the epistasis network, with the gene names, the effect size (Delta.Nd), a qualitative classification of the interaction, and the associated p- and q-values. The network can be plotted and manipulated by manually loading the merged table in Cytoscape.^5^ Cytoscape also provides basic functionality for network analysis through a convenient graphical interface. Alternatively, users may choose python-based libraries for network analysis and visualization, such as NetworkX and igraph. Finally, the modules of the epistasis network can be obtained with SiMap,^6^ a community detection tool that takes into account the sign and weight of the links.

## Limitations

**Figure 4:**
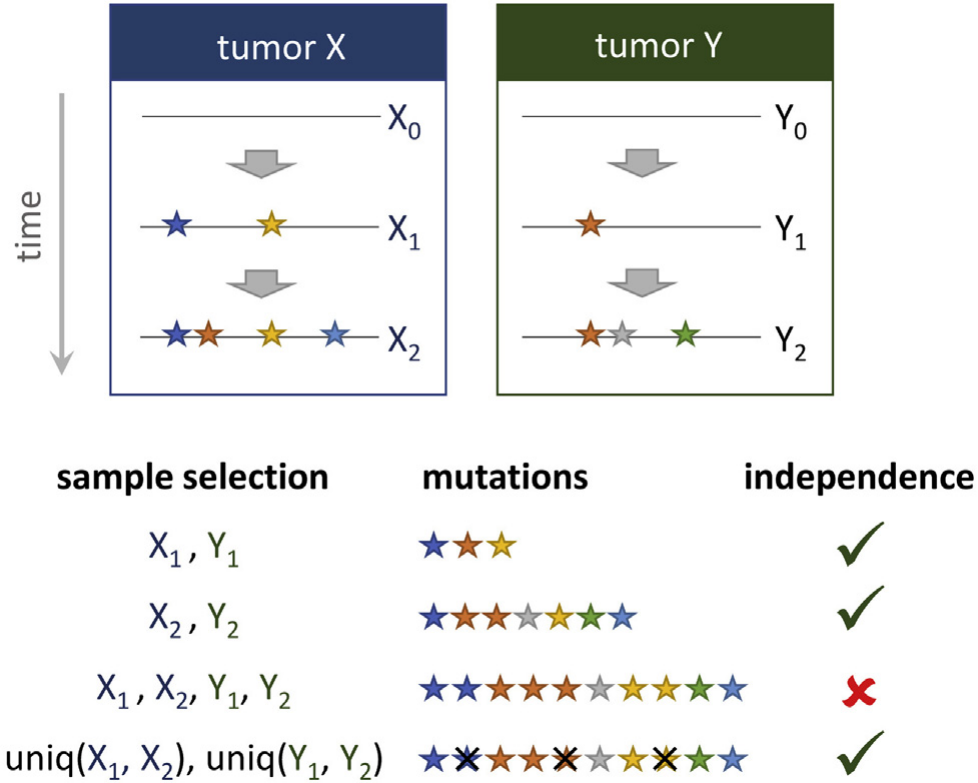
Schematic assessment of mutation independency Somatic mutations in two samples collected from different tumors always correspond to independent mutation events. However, redundant mutations found in different samples of the same tumor are probably the result of a single mutation event that occurred in a common ancestor. To deal with the latter, a possibility is to combine all the mutations found in samples from the same lineage, eliminating redundancy. Note that, in that case, the units of analysis will be the lineages (defined by the merging criteria), not the individual samples. In consequence, effect sizes should be interpreted as the number of drivers per lineage, not per sample. In the bottom row, uniq() stands for “unique”, that is, single representatives on a list of (possibly redundant) mutations.

*Coselens* infers mutational models directly from the data by assuming that each mutation in the dataset corresponds to a unique mutational event and there are no missing mutations. The first assumption is violated if samples are not independent; the second assumption fails if there are reversions and double mutations. Because of these limitations, *Coselens* is best suited to analyze somatic mutations and data from experimental evolution, for which independently evolved samples are available. Datasets including samples that are not fully independent can still be analyzed, but should be curated to remove redundant mutations if they are synapomorphic (see Figure 4 and troubleshooting 2).

*Coselens* was designed to study driver mutations. Accordingly, the reported effect sizes are most easily interpreted in a context of positive selection, where the excess of mutations indicates the average number of drivers in a gene. If this number is sufficiently small (around 0.2 or smaller) or if it can be assumed that each gene can harbor at most 1 driver mutation, the effect size can be also be interpreted as the probability of observing driver mutations in a gene. In contrast, finding more than 1 driver mutation per gene could be indicative of clonal interference, that is, the coexistence of multiple drivers that compete for fixation in a heterogeneous population. In scenarios (or genes) dominated by purifying selection, a negative number of drivers indicates the average number of mutations that have been purged by selection. Unlike with positive selection, it is not evident that the absolute number of purged mutations is more suitable for comparative purposes than a relative measure such as the dN/dS. The interpretation can be especially challenging if the group with fewer purged mutations has also a smaller mutational load. Therefore, users should be cautious when interpreting effect sizes involving strong purifying selection.

The sensitivity of *Coselens* depends on the total number of mutations observed in each group after pooling all the samples. As a general rule, the more mutations the higher the sensitivity. However, the presence of ultrahypermutators whose mutational signatures span more than 3 nucleotides or that accumulate non-negligible numbers of double mutations can lead to inaccurate estimates. To avoid that, it is advisable to remove heavily mutated samples from the analysis. In our experience with cancer somatic mutation data, a total of 250 mutations per Mb per group (2 × 7.5k mutations for whole exome analyses) is typically enough to detect significant effect sizes of the order of 0.1 driver mutation per gene per sample.

**Figure 5:**
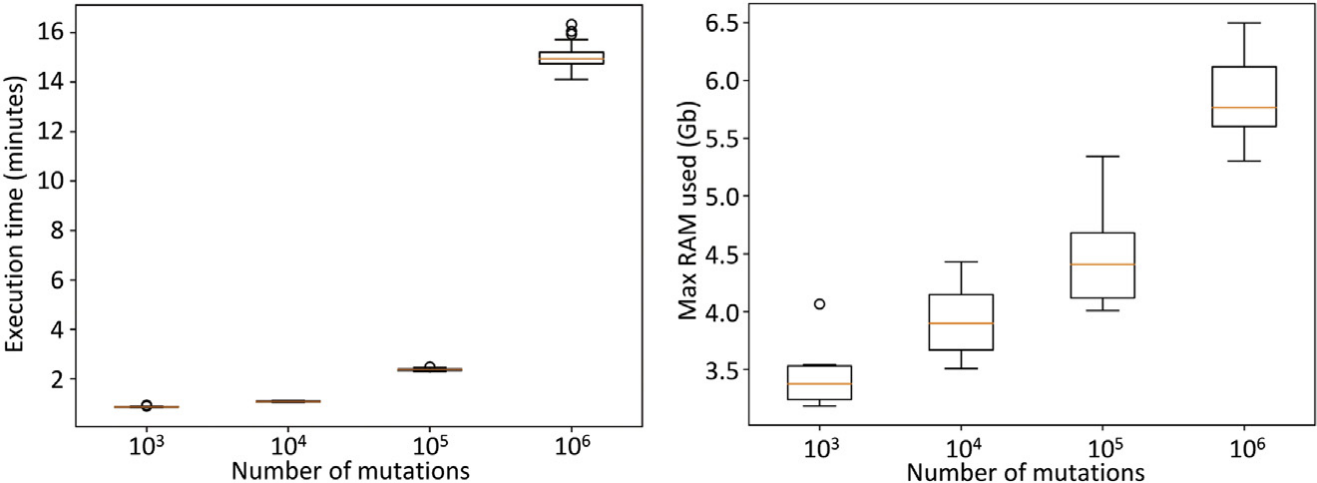
Runtime (left) and memory requirements (right) Each boxplot corresponds to 100 runs of Coselens using random datasets of different sizes (x-axis). Orange lines indicate the median, boxes cover the Q1-Q3 range, whiskers extend to the highest and lowest data point not considered outliers, circles represent outliers.

For small and medium-sized datasets (up to 100K mutations), *Coselens* typically runs in less than 5 min and requires less than 6Gb RAM, what makes it suitable for most personal laptops. With larger datasets, the runtime increases notably (around 15 min for 1M mutations and 1 h for 3M mutations), whereas the increase in memory with sample size is less pronounced (Figure 5).

## Troubleshooting

### Problem 1

In step 1, an error is produced when trying to load *Coselens*:

## Error in library("coselens") : there is no package called ‘coselens’

### Potential solution

Make sure that Coselens has been correctly installed (see section installation of Coselens and its dependencies). If automatic installation of dependencies produces an error, try manually installing them with the following commands:

>install.packages("devtools")

>install.packages("geometry")

>install.packages("seqinr")

>if (!require("BiocManager", quietly = TRUE)) install.packages("BiocManager")

>BiocManager::install("Iranges")

>BiocManager::install("Biostrings")

>BiocManager::install("GenomicRanges")

>install.packages("MASS")

If problems persist, it could be due to incompatibility among package versions. *Coselens* has been successfully tested with the following package versions: R version 4.2.2; devtools version 2.4.3; geometry version 0.4.6.1; seqinr version 4.2-23; GenomicRanges version 1.50.2; Biostrings version 2.66.0; IRanges version 2.32.0; and MASS version 7.3-58.

### Problem 2

In step 4, *Coselens* displays the following warning:

## Warning in dndscv(mutations): Same mutations observed in different

## sampleIDs. Please verify that these are independent events and remove

## duplicates otherwise.

### Potential solution

The warning is displayed if the same mutation is observed in different samples. This could indicate lack of independence between samples (for example, if the same tumor was sequenced multiple times). Repeated mutations can also result from convergent evolution in independent samples, especially in loci that behave as mutational hotspots. When running *Coselens*, assessing independence among samples is important because lack of independence affects the inference of the underlying mutation model.

• If coincident mutations in your dataset result from independent mutation events, you can safely ignore the warning.
• Otherwise, the mutation table should be curated to remove redundancy. A possible strategy in that case is to combine related samples and keep only one instance of each mutation (Figure 4). Because effect sizes depend on the average number of drivers per gene per sample, combining samples may have an impact on the estimated effect sizes. However, if combinations of samples are biologically meaningful (e.g., multiple samples from the same tumor), the effect sizes can still be interpreted in terms of the composite sampling unit (e.g., drivers per tumor instead of per sample).

### Problem 3

In step 4, *Coselens* produces an error:

## Error in coselens::dndscv[…]

## XX% mutations have a wrong reference base. Please confirm that you are not

## running data from a different assembly or species.

This error occurs if there are large inconsistencies between the reference bases in the input table and the reference database. The most likely cause is that the assembly used to build the input mutation table does not coincide with the default used by coselens (GRCh37/hg19) or with the reference database provided with the option refdb. In particular, this is the case if working with non-human data and not providing an adequate reference database. Although less likely, this error could also be caused by a poorly formatted input table (see data preparation – step 3).

If the number of inconsistencies between the input table and the reference database is small, a warning (instead of an error) is produced:

## Warning in coselens::dndscv[…] : XX% mutations have a wrong reference base

## (see the affected mutations in dndsout$wrongmuts). Please identify the causes

## and rerun dNdScv.

### Potential solution

To use *Coselens* with different species or assemblies, an alternative reference database (RefCDS object) must be provided with the option refdb. Moreover, the default covariates used by *Coselens* are specific to the GRCh37/hg19 assembly of the human genome. Therefore, to analyze data from other species, *Coselens* should be run either without covariates (option cv = NULL) or using covariates specifically generated for the species of interest. Covariate matrices should be formatted as data frames with genes as row names and one column per covariate. Custom covariate matrices can be directly provided to *Coselens* (e.g., cv = covariates) or saved and later retrieved from a .RDS file (e.g., cv = "/path/to/covariates_file.rds").

Precomputed reference databases and covariates for a few species and assemblies can be downloaded from https://github.com/im3sanger/dndscv_data/tree/master/data. More generally, alternative reference databases and covariates can be generated using the *dndscv* package. The procedure is explained in http://htmlpreview.github.io/?http://github.com/im3sanger/dndscv/blob/master/vignettes/buildref.html.

### Problem 4

In step 4, *Coselens* displays one of the following errors while running dNdScv:

## Error in coselens::dndscv[…] : Row names in the covariates matrix do not

## coincide with the gene names in the reference database. Please, provide a

## compatible covariates matrix or run dNdScv with the option cv=NULL.

This error is often displayed together with a warning:

## Warning in sapply(…) : longer object length is not a multiple of shorter object

### Potential solution

This error indicates that the covariates matrix is incompatible with the reference database. If analyzing non-human data or assemblies other than GRCh37/hg19, make sure that you provide compatible covariates or set cv = NULL to run *Coselens* without covariates. See troubleshooting 3 for further details on how to generate custom covariates.

### Problem 5

In step 4, *Coselens* displays the following warning:

## Note: XXX samples excluded for exceeding the limit of mutations per sample

## (see the max_coding_muts_per_sample argument in dndscv). XXX samples left

## after filtering.

### Potential solution

The warning is displayed if there are samples with >3,000 mutations. By default, *Coselens* identifies ultrahypermutated samples and removes them from the analysis, as they could lead to inaccurate estimates. If you are confident that hypermutation is not an issue in your data, you can override this behavior by adding the argument max_coding_muts_per_sample = Inf when calling *Coselens*.

### Problem 6

In step 4, the following error is produced when trying to run *Coselens*:

## Error in coselens(group1, group2, subset.genes.by = cancer_genes) :

## could not find function "coselens"

### Potential solution

The most likely cause of this error is that the *Coselens* package has not been loaded. Before testing for conditional selection, load the *Coselens* package:

>library("coselens")

If you get an error while trying to load the package, check troubleshooting 1.

### Problem 7

In step 4, an error is produced when trying to run *Coselens* on targeted sequencing data.

## Error in coselens::dndscv[…]

## The following input gene names are not in the RefCDS database.

### Potential solution

This error is produced when some genes in the list of sequenced genes are not present in the reference database. Possible causes could be misspelling of gene names in the sequenced gene list, use of alternative (synonymous) gene names that do not coincide with those in the reference database, or the use of an incompatible reference database (troubleshooting 3). The error message includes the list of missing gene names, which could help identify the source of the problem. To get the list of genes in the default reference database, run.

>data("dndscv_data_refcds_hg19", package="coselens")

>gene.list = sapply(RefCDS,function(x) x$gene_name)

### Problem 8

In step 4, *Coselens* displays some of the following warnings:

## In glm.fit : fitted rates numerically 0 occurred.

## In glm.fit : algorithm did not converge.

## In theta.ml(Y, mu, sum(w), w, limit = control$maxit, trace = control$trace > :

## iteration limit reached.

### Potential solution

These warnings indicate that the model fitting algorithm failed to converge, which potentially affects the reliability of the maximum likelihood estimates and the subsequent inference of driver mutations. In most cases, this problem arises because the number of mutations in some regions of the genome is too small to estimate the model parameters. This could happen if you are using targeted sequencing data but did not provide the list of sequenced genes when calling *Coselens* (see data preparation – step 4 and running Coselens – steps 3 and 4). Otherwise, it could mean that your dataset is too sparse or small. In that case, the ideal solution would be to increase the sample size. If that is not possible, an alternative option would be to use simpler substitution models, that require fitting fewer parameters. Note, however, that using simplistic mutation models can bias the estimates of positive and negative selection, at least in the case of cancer genomes.^2^ To use a substitution model with 12 rates (without trinucleotide context-dependence), install the *dndscv* package and run the following lines:

>data("submod_12r_3w", package="dndscv")

>coselens_out = coselens(group1, group2, sm = substmodel)

The first line loads the substitution model (substmodel) from the *dndscv* package and the second line runs *Coselens* with that substitution model. More information on using alternative substitution models can be found in the documentation of the *dndscv* package (https://github.com/im3sanger/dndscv).

### Problem 9

In step 5, the p-values in the summary table for substitutions and indels (coselens_out$overall_mut) are not significant, even though conditional selection for driver substitutions appears statistically significant in the substitutions table (coselens_out$substitutions) and effect sizes are substantial.

### Potential solution

The global p-values in coselens_out$overall_mut are calculated by applying Fisher’s formula to combine the p-values associated with substitutions (coselens_out$substitutions) and indels (coselens_out$indels). Fisher’s formula can be too conservative if the combined tests suffer from low sensitivity. In our experience with cancer somatic mutation data, the indel test only attains good sensitivity if the sample size is large (>100 samples) and indels are frequent. Otherwise, the low sensitivity of the indel test results in non-significant values of the global p-value despite significant levels of differential selection on substitutions. Thus, we recommend using the p-values from coselens_out$substitutions to assess significance of differential selection on substitutions, while restricting the use of coselens_out$overall_mut to datasets with large sample sizes and genes in which indels are the main subject of selection.

## Resource availability

### Lead contact

Further information and requests for resources and reagents should be directed to and will be fulfilled by the lead contact, Jaime Iranzo (jaime.iranzo@upm.es).

### Materials availability

This study did not generate new materials or unique reagents.

## Data Availability

The R implementation of the Coselens algorithm is publicly available at https://github.com/ggruenhagen3/coselens and a stable version has been deposited at https://zenodo.org/record/7343763. DOIs are listed in the key resources table. The example datasets used in this protocol are available at https://github.com/ggruenhagen3/coselens.
